# Early Postnatal Stress Impairs Cognitive Functions of Male Rats Persisting Until Adulthood

**DOI:** 10.3389/fnbeh.2018.00176

**Published:** 2018-08-17

**Authors:** Anna Holubová, Ivana Lukášková, Nikol Tomášová, Mária Šuhajdová, Romana Šlamberová

**Affiliations:** Department of Physiology, Third Faculty of Medicine, Charles University, Prague, Czechia

**Keywords:** methamphetamine, prenatal stress, postnatal stress, maternal separation, learning, memory

## Abstract

Methamphetamine (MA) is the most abused “hard” illicit drug in the Czech Republic. Drugs abused during pregnancy are not hazardous merely to the mother, but also to developing fetuses. The offspring of drug-addicted mothers are also often exposed to perinatal stressors that may impair brain development of affected progeny. The present study examines the effect of perinatal stressors and drug exposure on cognitive function in male progeny. In the present study, rat mothers were divided into three groups according to drug treatment during pregnancy: controls (C); saline (SA, s.c., 1 ml/kg); MA (s.c., 5 mg/ml/kg). Litters were divided into two groups according to postnatal stressors: non-stressed controls (N); Maternal separation (MS). For evaluation of learning and memory, adult male progeny were tested in the Morris Water Maze (MWM). Our results revealed no significant effects caused by prenatal drug or prenatal stress exposure. On the other hand, chronic postnatal stress, mediated by MS, significantly impaired learning on the Place Navigation test. In addition, MS was associated with changes in search strategies on the Place Navigation, Probe, and Memory Recall tests. Specifically, postnatal stress increased thigmotaxis, indicating less awareness of the hidden platform. In conclusion, the present study provides evidence that exposure to early postnatal stress significantly impairs cognitive functions of male rats, which persists into adulthood.

## Introduction

Methamphetamine (MA) is a highly addictive, illicit, psychomotor stimulant drug. MA, like other amphetamines, is a lipophilic molecule that distributes widely in an organism, including the CNS (Courtney and Ray, 2014). MA enhances monoamine release (especially dopamine, noradrenaline and serotonin) in the CNS and partially blocks their reuptake from synapses (Cho and Melega, 2002). Noradrenergic areas of interest include basal forebrain structures, the hippocampus and the prefrontal cortex. This is where behavioral changes such as excitation, memory consolidation and cognitive processes have been observed (Berridge and Waterhouse, 2003). Drug abuse during pregnancy is a growing, world-wide problem. Approximately 66% of drug addicts in Czech Republic abuse MA as their first drug of choice (Vavrinková et al., 2001). Moreover, statistical data from the US show that a considerable number of drug addicted women switch from other hard drugs to MA during their pregnancy (Marwick, 2000). The reason is assumed to be that MA reduces pregnancy-related weight gain and pregnancy-related fatigue. Drugs abused during pregnancy are not hazardous only to the mother; many drugs easily cross the placental membrane and cause changes in the developing fetus that can have long-lasting consequences (Šlamberová, 2012).

Experimental studies have shown that high doses of MA during gestation can have teratogenic effects and lead to malformations (Yamamoto et al., 1992; Acuff-Smith et al., 1996; Cho and Melega, 2002). Clinical studies have demonstrated that newborns of MA-addicted mothers are born with lower birth weights, smaller head circumferences, and postnatal functional development is impaired (Oro and Dixon, 1987; Little et al., 1988; Wouldes et al., 2004). Studies have also shown that prenatal MA exposure leads to subcortical brain structures with smaller volumes (i.e., putamen, globus pallidus, hippocampus and caudate nucleus) as well as an increase in related neurocognitive deficits, compared to controls. Reduction in the size of these brain structures correlates with reduced attention, delayed verbal memory (Chang et al., 2004), and altered adaptability to stress (Wouldes et al., 2004). Effects on behavior and cognitive function appear to be long-lasting (Hansen et al., 1993). Changes of the development and activity of the hippocampus have been shown in the offspring prenatally exposed to MA as well as to prenatal stress (Lemaire et al., 2000; Anacker et al., 2013; Bernaskova et al., 2017). Chronic prenatal exposure to MA changes the stress response by increasing secretion of glucocorticoids that activate glucocorticoid receptors (GR) through negative feedback earlier than normal (Williams et al., 2006; Anacker et al., 2013; Bernaskova et al., 2017). High concentrations of cortisol then through GR reduce proliferation and neuronal differentiation (Anacker et al., 2013). Moreover, MA administration enhances release of some neuromodulators (such as dopamine, norepinephrine and serotonin) into the synaptic cleft; this may significantly change the functional states of hippocampus (Thompson et al., 2009; Swant et al., 2010). Study Hori et al. (2015) suggested that chronic prenatal MA exposure changes the NMDA responses secondary to genetic changes of NMDA receptor. These modifications decrease the long-term potentiation and activity of CA1 hippocampal region that may further decrease cognition and learning ability (Bernaskova et al., 2017).

Apart from the direct effects on the child’s health, indirect effects must also be considered. Research has shown that drug addicted mothers often provide inadequate care for their newborns. Ninety percent of cases involve young, single mothers who are often unemployed and live in unsettled social conditions (Vavrinková et al., 2001). Combined with inadequate parental care, these conditions can cause psychic deprivation and mental retardation of affected children (Šlamberová and Charousová, 2008). Preclinical trials have shown the harmful effects of prenatal stress on physical development and development of cognitive functions. Women have naturally increased levels of cortisol during pregnancy, therefore, maternal stress during pregnancy can further increase the level of circulating cortisol as well as lower the expression and activity of protective enzymes in placenta, which can lead to less protected fetuses (Antonelli et al., 2017). An increased rate of anxiety and depression, learning difficulties and heightened reactivity to drugs are all possible behavioral repercussions in affected children.

Barrot et al. (2005) found that prenatal stress influences specific nuclei in the amygdala resulting in anxiogenic effects during testing in an elevated plus maze. The HPA axis is closely connected with the limbic system, which can modulate changes in emotions, cognition and personality. More specifically, prenatal stress in children has been associated with changes in learning and memory, behavior, physical and emotional problems, autism, and ADHD (attention deficit hyperactivity disorder; Charil et al., 2010; Antonelli et al., 2017). Project Ice Storm (Laplante et al., 2008) documented 89 young children that were prenatally exposed to a natural catastrophe (Ice Storm, Jan., 1998, Quebec, Canada). It confirmed that prenatal stress significantly and negatively affects cognitive functions (evaluated using Storm 32 questionnaires, Impact of Events Scale-Revised) and language abilities (Wechsler Preschool and Primary Scale of Intelligence-Revised and Peabody Picture Vocabulary Test-Revised) in five and half-year-old children (Laplante et al., 2008). Clinical studies by Lin et al. (2017) and Zhou et al. (2017) reported that prenatal stress negatively influences development of cognitive functions in toddlers. Furthermore, preclinical studies have shown that maternal injections during pregnancy, regardless of injected substance (drug or saline (SA)), induces long-lasting impairment on stress responsiveness in adult progeny (Peters, 1986; Šlamberová et al., 2002; Bernaskova et al., 2017). Therefore, drug injections, administered during pregnancy, can be used as a prenatal stressor.

MA used during pregnancy is also associated with poor maternal care during the lactation period (Šlamberová et al., 2005; Malinová-Ševčíková et al., 2014). Recent clinical studies have shown that maternal MA abuse increases maternal symptoms of depression and parenting stress (Liles et al., 2012). The affected progeny is therefore often ignored and exposed to postnatal stressors (Larsson et al., 1979). Maternal separation (MS) is a model that has been used in preclinical trials for over 50 years. This social stressor is based on repeated postnatal disruption of mother-pup interactions, which can lead to poor maternal care (Huot et al., 2004). MS can influence the HPA axis even during periods of low responsiveness, which occurs during the first 2 weeks of the postnatal period (Sapolsky and Meaney, 1986; Pryce and Feldon, 2003; Marais et al., 2008; Lajud et al., 2012). It is assumed that chronic increases in glucocorticoid levels, following long-term MS, could affect the hippocampus, which contains high concentrations of mineralocorticoids and GR. This may lead to a modification in the number of receptors and abnormalities of the HPA axis (Sapolsky, 1985; Huot et al., 2002) leading to maladaptive brain development and consequent cognitive impairment in adulthood (Andersen and Teicher, 2004; Aisa et al., 2009). It has been shown that early postnatal stress in rats induces changes in the hippocampus by increasing proliferation and differentiation of dentate gyrus cells and increasing the number of immature cells migrating postnatally from the dentate gyrus. In males, these changes manifest as a reduced score on learning and memory tests (Naninck et al., 2015). The early postnatal period is thus a very sensitive time for the developing brain, in both humans and rodents. Stressors during this period can lead to a decline in cognitive function and an impairment of emotional development (Krugers et al., 2017). Moreover, since maternal chronic stress during the lactation period can impair maternal nursing, affected progeny may lose the benefit of adequate breastfeeding, which has been shown to negatively affect general cognitive development and behavior (Nephew and Murgatroyd, 2013).

However, in our previous study, the group exposed to MS gained significantly more weight than non-stressed offspring, regardless of prenatal treatment (Holubová et al., 2017). One possible explanation could be decreased plasma leptin levels in rats following MS compared to controls (Salzmann et al., 2004; Walker et al., 2004; Schmidt et al., 2006; Friedman, 2011; Holubová et al., 2016), which stimulates the appetite (Friedman, 2011). This is further supported by behavior of pups affected MS that spend more time by feeding themselves from their mothers, i.e., stressed mothers breast-fed more in the passive position compared to controls. Meaning that, overall, the pups with MS fed more often than controls (non-stressed pups; Holubová et al., 2017). This is in agreement with a study by Mesquita et al. (2007) where feeding time of pups was significantly increased in MS groups, probably to compensate for long maternal absences (Mesquita et al., 2007). Additionally, food and water intake were monitored during the 2 days prior to analyses in our other study (Holubová et al., 2018) with adult male rats previously exposed to maternal stress or not (non-stressed controls). There were no significant differences in food intake among any of the groups of adult rats before the experiment (Holubová et al., 2018).

Our previous study suggests that a combination of maternal postnatal stressors with prenatal stress and drug exposure has detrimental effects on maternal behavior as well as on the sensorimotor development of their pups. MA exposure during pregnancy seemed to be the decisive factor for those impairments (Holubová et al., 2017). The present study examines the effect of long-term perinatal maternal stressors combined with prenatal drug exposure on cognitive functions such as learning and memory in adult male rats. We worked from the hypotheses that combination of prenatal MA and perinatal stress impairs learning ability and long-term memory retrieval, and that these effects can persist into adulthood. Testing used modified methods from our previous studies (Schutová et al., 2009; Holubová et al., 2016, 2017; Macúchová et al., 2017).

## Materials and Methods

The procedures for animal experimentation utilized in this study were reviewed and approved by the Institutional Animal Care and Use Committee of Charles University and were in agreement with the Requirements of the Czech Government under the Policy of Human Care of Laboratory Animals (No. 246/1992), with subsequent regulations of the Ministry of Agriculture of the Czech Republic (No. 311/1997).

### Animals and Prenatal Care

Forty-eight nulliparous adult, female, albino Wistar rats (250–300 g) were delivered by Velaz (Prague, Czechia) from Charles River Laboratories International, Inc. Females were housed in groups (4 per cage) and left undisturbed for 1 week in a temperature-controlled (22–24°C) room, with food and water *ad libitum*, on a 12 h light cycle (lights on at 6:00 am) to acclimatize. After 1 week, females were housed overnight with mature males. Females were then randomly divided into three groups according to daily treatments during gravidity; group (1) MA (5 mg/kg s.c.), group (2) SA (1 ml/kg s.c.) or group (3) C controls without injections. A dose of 5 mg/kg was chosen deliberately because it leads to the same concentration of MA in rat brains as that found in the fetal brains of drug addicted women (Acuff-Smith et al., 1996). On day 20 of gestation, females were placed in maternity cages (1 per cage). The day of delivery was counted as postnatal day (PD) 0.

### Postnatal Care

On PD 1 litter size was adjusted to 12 pups. Whenever possible, equal numbers of males and females were raised by each mother. The pups were divided so that one mother raised four pups from MA, four pups from SA, and four pups from control mothers. For recognition, prenatally MA-exposed pups were intradermally injected with India ink in the left hind paw, SA-exposed pups in right hind paw, and controls were not tattooed. Pups were then divided into two groups: controls (N) and maternal separation (S).

#### Social Stressor

MS, as a social stressor, was conducted daily (PD 1–21) for 3 h per day, between 8:00 am and 11:00 am (Plotsky et al., 2005; Lajud et al., 2012; Holubová et al., 2016, 2018). Pups from group (S) were gently removed from their maternity cage and placed in a separate cage in another room; the mothers were left undisturbed in their home cages. The separate cage with pups was always placed on a heating pad to prevent chilling. After 3 h of separation, the pups were returned to their home cages. The non-stressed controls (N) were left undisturbed during the lactation period.

### The Morris Water Maze Test

Only male progeny was used to evaluate cognitive function (Morris Water Maze test, MWM); each group with combination of prenatal and postnatal treatments involved eight subjects. Adult male progeny, 60–80 days of age, were tested over a 12-day period. Based on results from previous studies, this study used three specific tests (Schutová et al., 2009; Macúchová et al., 2017): (1) the Place Navigation test; (2) the Probe test; and (3) the Memory Retention test, all of which used a MWM. The water maze consisted of a circular tank (diameter = 2 meters) with water at 22°C ± 2.5°C. A transparent platform was hidden in the tank, 1.5 cm below the water surface, making it invisible to the rats during testing. Trials were recorded using the EthoVision XT 10 video-tracking system.

#### The Place Navigation Test

During the first 6 days of the experiment, animals were trained to find the hidden platform starting from four different positions located around the perimeter of the MMW tank (i.e., North, South, East and West). If the animal did not find the platform with the 60 s limit, it was gently guided to the platform by the experimenter. The position of platform was the same throughout this part of the experiment. Eight trials, i.e., two rounds from the North, South, East and West starting positions, were performed per day. At the end of each trial, the animal left undisturbed on the platform for 30 s prior to starting the next trial. This was done to facilitate learning the platform’s position as well as the platform’s position relative to external cues in the room. After the eighth trial on an experimental day, animals were dried and returned to their home cages. The measured parameters during the trials were: (1) search error (the cumulative distance from the hidden platform) [cm]; (2) distance traveled (the cumulative length of the swim-path in the tank) [cm]; (3) latency of platform acquisition [s]; and (4) swimming velocity [cm/s]. A fifth parameter, search strategy, was also evaluated. Two search strategies were analyzed: thigmotaxis and scanning. Evaluation of search strategies was modified from our previous studies (Schutová et al., 2009; Macúchová et al., 2017) to allow digital analysis using the EthoVision XT 10 system. Thigmotaxis was defined as swimming along the perimeter of the tank, i.e., within 30 cm of the wall of the tank; while scanning was defined as swimming in the area 1 m in the diameter with the platform in the center. Both strategies were counted as the amount of time spent in the given areas and the percentage of total time spent using the strategy.

#### The Probe Test

The Probe test was run on the 8th day. The platform was removed from the tank and the animal was placed in the “North” position and allowed to swim for 60 s. The followed parameters were measured: distance traveled [cm]; velocity of swimming [cm/s]; and both search strategies (thigmotaxis and scanning).

#### The Retention Memory Test

The Retention Memory test was conducted on the 12th day (final day). Each animal had to find the hidden platform located in the same position it occupied during the Place Navigation test. Each animal completed eight trials, with each trial lasting 60 s, just as in the Place Navigation test. All the same parameters and strategies were analyzed as in the Place Navigation test.

### Statistical Analysis

Two-way ANOVA (Prenatal exposure × Postnatal stress) with repeated measure (days × trials/day) was used to analyze the data from the Place Navigation test. Similarly, two-way ANOVA (Prenatal exposure × Postnatal stress) was used to analyze the data from the Retention Memory test. The Probe test data was analyzed by ANOVA where between factors were: Prenatal exposure × Postnatal stress. Bonferroni’s test was used for *post hoc* test comparisons. Differences were considered significant if *p* < 0.05.

## Results

### Effect of the Prenatal Drug (MA) and Stress (SA) Exposure

There were no significant differences among the tested groups in any of the analyzed parameters or on any of the tests (i.e., Place Navigation, Probe, or Memory Recall test). Although no significant effect in traveled distance (*F*_(2,42)_ = 0.88; *p* = 0.42), latency of platform acquisition (*F*_(2,40)_ = 1.21; *p* = 0.31), and search error (*F*_(2,42)_ = 1.91; *p* = 0.16) was found in either prenatally treated groups (SA and MA), there were obvious tendencies for these groups to perform worse compared to controls on the Place Navigation test as well as in the Memory Recall test (figures not shown). Similarly, no significant effects of prenatal MA or SA exposure was found relative to the percentage of time spent in the two search strategies, i.e., thigmotaxis (*F*_(2,42)_ = 2.25; *p* = 0.12) vs. scanning (*F*_(2,42)_ = 1.76; *p* = 0.18); however, search strategy tendencies suggested worse performance by the SA/MA groups compared to controls.

### Effect of the Postnatal Stress Exposure

The main effect of postnatal stress was demonstrated between groups of postnatally stressed males and unstressed controls. The measurement of search strategies led to comparable results in all three of the following tests.

#### The Place Navigation Test

**Figure 1:**
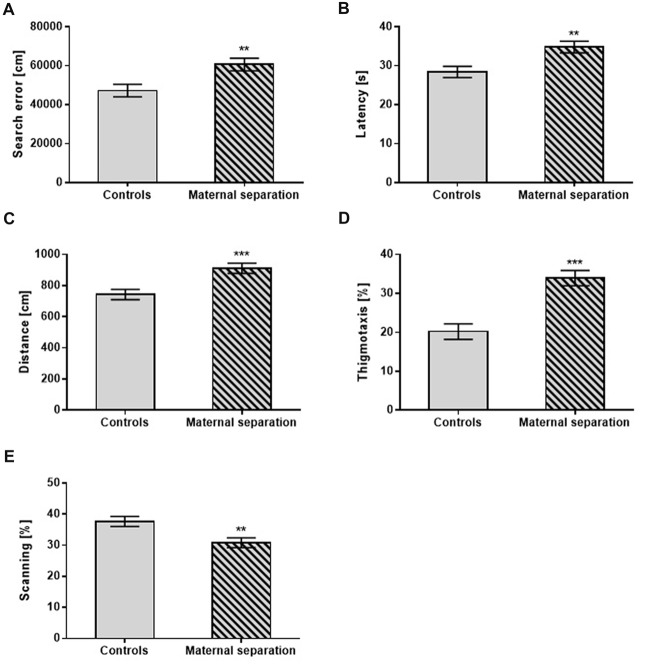
The effect of Maternal separation (MS) on the Place Navigation test. **(A)** Search error, **(B)** Latency, **(C)** Distance, **(D)** Thigmotaxis, **(E)** Scanning. Values are means ± SEM. **p < 0.01; ***p < 0.001. Significant differences were seen between groups stressed by MS and controls, regardless of prenatal treatment.

Early postnatal exposure to MS significantly increased search error (*F*_(1,42)_ = 8.21; *p* < 0.01), traveled distance (*F*_(1,42)_ = 14.28; *p* < 0.001) and latency in reaching the hidden platform (*F*_(1,40)_ = 7.16; *p* < 0.01) compared to unstressed controls. In search strategies, males with MS spent a higher percentage of time in thigmotaxis (*F*_(1,42)_ = 22.52; *p* < 0.001) and less time in scanning (*F*_(1,42)_ = 11.33; *p* < 0.01) than unstressed controls regardless, of prenatal treatment (Figure 1).

#### The Probe Test

**Figure 2:**
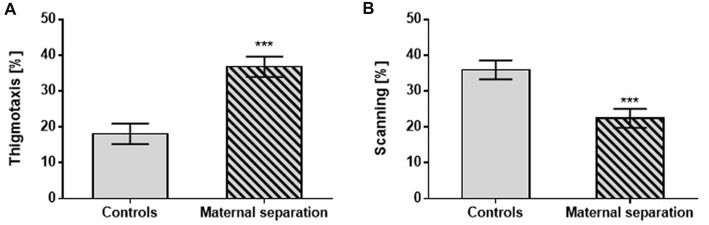
The effect of MS on the Probe test. **(A)** Thigmotaxis, **(B)** Scanning. Values are means ± SEM. ***p < 0.001. A significant difference was seen between groups stressed by MS and controls, regardless of prenatal treatment.

The Probe Test did not reveal any differences between controls and postnatally stressed male rats in any of the measured parameters. However, significantly different performance by postnatally stressed relative to unstressed controls was seen in search strategies: thigmotaxis (*F*_(1,42)_ = 25.40; *p* < 0.001) vs. scanning (*F*_(1,42)_ = 13.07; *p* < 0.001), regardless of prenatal exposure (Figure 2).

#### The Memory Recall Test

**Figure 3:**
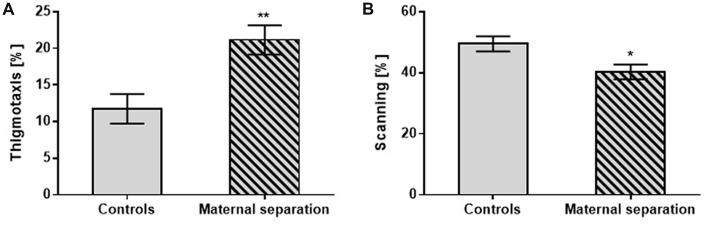
The effect of MS on the Memory Recall test. **(A)** Thigmotaxis, **(B)** Scanning. Values are means ± SEM. *p < 0.05; **p < 0.01. Significant differences were seen between groups stressed by MS and controls, regardless of prenatal treatment.

On the Memory Recall test, as with the other tests, the effect of postnatally stressed adult offspring manifested in changes in search strategies: thigmotaxis (*F*_(1,42)_ = 11.07; *p* < 0.01) vs. scanning (*F*_(1,42)_ = 11.44; *p* < 0.05) compared to unstressed controls (Figure 3). The measurement of other parameters found no significant changes between stressed and unstressed groups.

## Discussion

### Effect of the Prenatal Methamphetamine and Stress Exposure

The first goal in the present study was to determine the effect of prenatal drug (MA) and stress (SA) exposure on the learning abilities of adult male rats. No significant effects from prenatal MA or SA exposure on learning and memory in the MWM were found compared to controls in the present study. However, it should be mentioned that rats affected by prenatal stress or drug exposure tended to perform worse compared to controls in all tests (i.e., Place Navigation, Probe and Memory Recall tests). Some preclinical studies suggest that chronic application of MA during pregnancy can influence development of the CNS of fetuses (Šlamberová et al., 2006), specifically the striatum and hippocampus, which are areas important for spatial learning and memory in both humans and rodents (McDonald and White, 1994). Additionally, study Šlamberová et al. (2005) found significantly reduced active nursing in MA-treated mothers relative to control rats. There is evidence showing that insufficient breastfeeding may affect cognitive development and behavior of infants (Nephew and Murgatroyd, 2013). However, some following studies (Schutová et al., 2009; Hrubá et al., 2010) as well as the present study did not show significant impairment of cognitive functions in adult offspring prenatally exposed to MA (evaluated by MWM). Furthermore, study in Holubová et al. (2017) did not reveal significant differences in active nursing behavior of rat-mothers with same treatments as in Šlamberová et al.’s (2005) study.

Results of studies examining prenatal exposure to MA on cognitive functions in adulthood are contradictory. These differences apparently relate to differences in dosage and length of drug exposure (Schutová et al., 2009) used in the various studies. Acuff-Smith et al. (1996) uncovered the influence of MA applied prenatally on the cognitive function of offspring relative to the stage of the pregnancy in which the MA was administered. High doses (15–20 mg/kg) administered in the beginning of gestation caused a deterioration in spatial memory which could be observed using the MWM. Conversely, lower doses (5–10 mg/kg) had no impact on cognitive functions in adult male rats using the same tests (Acuff-Smith et al., 1996; Schutová et al., 2009). The dose of MA used in this study was 5 mg/kg, which is the standard dose used in our experiments (Šlamberová et al., 2005; Schutová et al., 2009) and is known to produce fetal brain drug concentrations similar to those found in fetuses of drug-abusing women (Acuff-Smith et al., 1996; Rambousek et al., 2014). Our results, i.e., no significant effects, is thus in line with other previous studies.

Moreover, although prenatal exposure to MA does not impact learning and memory, postnatal exposure to MA shows impairments of cognition (Hrubá et al., 2010). As authors (Hrubá et al., 2010) concluded, the study indicates that postnatal but not prenatal exposure to MA affects learning in adult male rats. In addition, Bayer et al.’s (1993) study showed that the hippocampus in rats is still developing during the PDs 11–20 which could be analogous to human hippocampal development during the third trimester of pregnancy. Vorhees et al.’s (1994) study demonstrated that postnatal MA exposure during PD 11–20 affects spatial learning and memory in rats evaluated by MWM. Williams et al.’s (2003) study demonstrated that neonatal exposure to MA at daily doses of 5, 10 or 15 mg/kg administrated from PD 11–20 produced lasting spatial learning and memory deficits. The early postnatal period may be thus more critical for cognitive functions in rats than the prenatal period. Nevertheless, in humans, clinical studies suggested that prenatal MA exposure leads to long-term cognitive deficits (Smith et al., 2001; Chang et al., 2004).

### Effect of the Postnatal Stress Exposure

The second goal of the present study was to determine the effect of postnatal (days 1–21 after birth) stress exposure. In our study, the effects of postnatal stress exposure were measured using three different MWM tests. The Place Navigation test showed that early postnatal stress exposure significantly increased the measured parameters (i.e., search error, traveled distance, latency to search hidden platform, and search strategies) compared to controls. The Probe test found significant changes in search strategies that further supported the negative impact of early adverse experiences. Finally, the Memory Recall test found differences in search strategies suggesting that retrieval of long-term memory was affected by early postnatal stress. Recent preclinical studies support our results, in that early adverse experiences may affect brain structures and functions, which could increase vulnerability to cognitive and psychiatric disorders (Heim and Nemeroff, 2001; Plotsky et al., 2005; Aisa et al., 2007). Aisa et al. (2009) found that early postnatal stress had long-lasting effects on hippocampal structure and function. The hippocampus is crucial in long-term memory formation and spatial learning (Morris et al., 2003; Mirescu et al., 2004) and is also easily influenced by stress hormones during early development (de Kloet et al., 1999). Granule cell development in the dentate gyrus of the rat hippocampus starts during late embryogenesis and continues during the first postnatal weeks (Altman and Bayer, 1990), as such, stress caused by MS during the early PDs can affect the normal development of hippocampal structures (Mirescu et al., 2004; Oreland et al., 2010).

Cao et al. (2014) investigated the influence of timing of early MS on cognitive functions in male rats. The study compared two periods of MS, i.e., PD 2–9 and PD 14–21. The results revealed that the “escape latency” in the MS group PD 14–21, was seriously impaired compared with controls (in a MWM test), while there was no significant difference between MS group PD 2–9 and controls. These results indicate that the critical period of rat hippocampus development may be PDs 14–21 (Galvan et al., 2000; Kolb and Gibb, 2011). According to a clinical study of Stevenson-Hinde (Stevenson-Hinde, 2007), a harmonious mother-infant relationship is key for normal development of a child’s brain; the crucial period is from age of 6- to 18-months. Early MS can lead to impairments of attention, memory, reasoning ability and psychological status in children (Cao et al., 2014).

According to Choy et al. (2008), altered expression of brain-derived neurotrophic factor (BDNF) is implicated in the etiology of many psychiatric illnesses, including schizophrenia, and may explain certain cognitive deficits. Choy’s study examined the long-term effect of early stress (maternal deprivation) and late stress (chronic young-adult treatment with stress hormones) and combination of both on BDNF expression in the hippocampus of rats. Learning, memory, short term spatial memory, long term spatial memory and working memory were tested. The results showed that “two-hit” rats had a significant reduction in BDNF expression in the dentate gyrus. These rats lagged during long-term spatial memory tests, learning tests and memory tests. Cognitive functions were also altered in the other two exposed groups. This study showed the significant impairing effects of early and late stress on cognitive functions in rats (Choy et al., 2008).

Overall, it appears that early postnatal stress can strongly impact the development of cognitive abilities in rats. On the other hand, prenatal drug and stress exposure did not revealed significant changes in MWM tasks. To the best of our knowledge, there is no other study evaluating the combination of these perinatal factors that could be presented in offspring of drug addicted mothers. The early postnatal stress seems to be more critical for cognitive functions than prenatal factors such as drug exposure (MA injections) or daily stress (SA injections) during pregnancy. Moreover, according to our results, the detrimental effects of early postnatal stress on learning and memory could have a significant effect well into adulthood. Nevertheless, more studies with distinct setups are needed to determine the mechanism of the impairment of neurological development in offspring exposed to prenatal drug exposure and MS.

## Author Contributions

AH as PhD student is responsible for undergraduate student IL, NT and MŠ. She is responsible for the experimental part as well as for the present manuscript. IL, NT and MŠ has been involved in the experimental part as well as in writing the manuscript. RŠ is a supervisor of AH and head of the department and the laboratory where this study has been conducted. She is integral to the present study.

## Conflict of Interest Statement

The authors declare that the research was conducted in the absence of any commercial or financial relationships that could be construed as a potential conflict of interest.

## References

[B1] Acuff-Smith K. D. Schilling M. A. Fisher J. E. Vorhees C. V. (1996). Stage-specific effects of prenatal d-methamphetamine exposure on behavioral and eye development in rats. Neurotoxicol. Teratol. 18, 199–215. 10.1016/0892-0362(95)02015-2 8709932

[B2] Aisa B. Elizalde N. Tordera R. Lasheras B. Del Rio J. Ramirez M. J. (2009). Effects of neonatal stress on markers of synaptic plasticity in the hippocampus: implications for spatial memory. Hippocampus 19, 1222–1231. 10.1002/hipo.20586 19309038

[B3] Aisa B. Tordera R. Lasheras B. Del Rio J. Ramirez M. J. (2007). Cognitive impairment associated to HPA axis hyperactivity after maternal separation in rats. Psychoneuroendocrinology 32, 256–266. 10.1016/j.psyneuen.2006.12.013 17307298

[B4] Altman J. Bayer S. A. (1990). Migration and distribution of two populations of hippocampal granule cell precursors during the perinatal and postnatal periods. J. Comp. Neurol. 301, 365–381. 10.1002/cne.903010304 2262596

[B5] Anacker C. Cattaneo A. Luoni A. Musaelyan K. Zunszain P. A. Milanesi E.. (2013). Glucocorticoid-related molecular signaling pathways regulating hippocampal neurogenesis. Neuropsychopharmacology 38, 872–883. 10.1038/npp.2012.253 23303060 PMC3672002

[B6] Andersen S. L. Teicher M. H. (2004). Delayed effects of early stress on hippocampal development. Neuropsychopharmacology 29, 1988–1993. 10.1038/sj.npp.1300528 15316569

[B7] Antonelli M. C. Pallarés M. E. Ceccatelli S. Spulber S. (2017). Long-term consequences of prenatal stress and neurotoxicants exposure on neurodevelopment. Prog. Neurobiol. 155, 21–35. 10.1016/j.pneurobio.2016.05.005 27236051

[B8] Barrot M. Wallace D. L. Bolaños C. A. Graham D. L. Perrotti L. I. Neve R. L.. (2005). Regulation of anxiety and initiation of sexual behavior by CREB in the nucleus accumbens. Proc. Natl. Acad. Sci. U S A 102, 8357–8362. 10.1073/pnas.0500587102 15923261 PMC1149417

[B9] Bayer S. A. Altman J. Russo R. J. Zhang X. (1993). Timetables of neurogenesis in the human brain based on experimentally determined patterns in the rat. Neurotoxicology 14, 83–144. 8361683

[B10] Bernaskova K. Tomkova S. Slamberova R. (2017). Are changes in excitability in the hippocampus of adult male rats induced by prenatal methamphetamine exposure or stress? Epilepsy Res. 137, 132–138. 10.1016/j.eplepsyres.2017.08.009 28886886

[B11] Berridge C. W. Waterhouse B. D. (2003). The locus coeruleus-noradrenergic system: modulation of behavioral state and state-dependent cognitive processes. Brain Res. Rev. 42, 33–84. 10.1016/s0165-0173(03)00143-7 12668290

[B12] Cao X. Huang S. Cao J. Chen T. Zhu P. Zhu R.. (2014). The timing of maternal separation affects morris water maze performance and long-term potentiation in male rats. Dev. Psychobiol. 56, 1102–1109. 10.1002/dev.21130 23712516

[B13] Chang L. Smith L. M. Lopresti C. Yonekura M. L. Kuo J. Walot I.. (2004). Smaller subcortical volumes and cognitive deficits in children with prenatal methamphetamine exposure. Psychiatry Res. 132, 95–106. 10.1016/j.pscychresns.2004.06.004 15598544

[B14] Charil A. Laplante D. P. Vaillancourt C. King S. (2010). Prenatal stress and brain development. Brain Res. Rev. 65, 56–79. 10.1016/j.brainresrev.2010.06.002 20550950

[B15] Cho A. K. Melega W. P. (2002). Patterns of methamphetamine abuse and their consequences. J. Addict. Dis. 21, 21–34. 10.1300/j069v21n01_03 11831497

[B16] Choy K. H. De Visser Y. Nichols N. R. van den Buuse M. (2008). Combined neonatal stress and young-adult glucocorticoid stimulation in rats reduce BDNF expression in hippocampus: effects on learning and memory. Hippocampus 18, 655–667. 10.1002/hipo.20425 18398848

[B17] Courtney K. E. Ray L. A. (2014). Methamphetamine: an update on epidemiology, pharmacology, clinical phenomenology, and treatment literature. Drug Alcohol. Depend. 143, 11–21. 10.1016/j.drugalcdep.2014.08.003 25176528 PMC4164186

[B18] de Kloet E. R. Oitzl M. S. Joëls M. (1999). Stress and cognition: are corticosteroids good or bad guys? Trends Neurosci. 22, 422–426. 10.1016/s0166-2236(99)01438-1 10481183

[B19] Friedman J. M. (2011). Leptin and the regulation of body weigh. Keio J. Med. 60, 1–9. 10.2302/kjm.60.1 21460597

[B20] Galvan C. D. Hrachovy R. A. Smith K. L. Swann J. W. (2000). Blockade of neuronal activity during hippocampal development produces a chronic focal epilepsy in the rat. J. Neurosci. 20, 2904–2916. 10.1523/jneurosci.20-08-02904.2000 10751443 PMC6772221

[B21] Hansen R. L. Struthers J. M. Gospe S. M. Jr. (1993). Visual evoked potentials and visual processing in stimulant drug-exposed infants. Dev. Med. Child Neurol. 35, 798–805. 10.1111/j.1469-8749.1993.tb11731.x 8354431

[B22] Heim C. Nemeroff C. B. (2001). The role of childhood trauma in the neurobiology of mood and anxiety disorders: preclinical and clinical studies. Biol. Psychiatry 49, 1023–1039. 10.1016/s0006-3223(01)01157-x 11430844

[B23] Holubová A. Mikulecká A. Pometlová M. Nohejlová K. Slamberová R. (2018). Long-term early life adverse experience impairs responsiveness to exteroceptive stimuli in adult rats. Behav. Processes 149, 59–64. 10.1016/j.beproc.2018.02.005 29438728

[B24] Holubová A. Ševčíková M. Macúchová E. Hrebíčková I. Pometlová M. Šlamberová R. (2017). Effects of perinatal stress and drug abuse on maternal behavior and sensorimotor development of affected progeny. Physiol. Res. 66, S481–S491. 29355375 10.33549/physiolres.933800

[B25] Holubová A. Štofková A. Jurčovičová J. Šlamberová R. (2016). The effect of neonatal maternal stress on plasma levels of adrenocorticotropic hormone, corticosterone, leptin and ghrelin in adult male rats exposed to acute heterotypic stressor. Physiol Res. 65, S557–S566. 28006938 10.33549/physiolres.933530

[B26] Hori N. Kadota T. Akaike N. (2015). Functional changes in piriform cortex pyramidal neurons in the chronic methamphetamine-treated rat. Gen. Physiol. Biophys. 34, 5–12. 10.4149/gpb_2014024 25367761

[B27] Hrubá L. Schutová B. Pometlová M. Rokyta R. Slamberová R. (2010). Effect of methamphetamine exposure and cross-fostering on cognitive function in adult male rats. Behav. Brain Res. 208, 63–71. 10.1016/j.bbr.2009.11.001 19900489

[B28] Huot R. L. Gonzalez M. E. Ladd C. O. Thrivikraman K. V. Plotsky P. M. (2004). Foster litters prevent hypothalamic-pituitary-adrenal axis sensitization mediated by neonatal maternal separation. Psychoneuroendocrinology 29, 279–289. 10.1016/s0306-4530(03)00028-3 14604606

[B29] Huot R. L. Plotsky P. M. Lenox R. H. Mcnamara R. K. (2002). Neonatal maternal separation reduces hippocampal mossy fiber density in adult Long Evans rats. Brain Res. 950, 52–63. 10.1016/s0006-8993(02)02985-2 12231228

[B30] Kolb B. Gibb R. (2011). Brain plasticity and behaviour in the developing brain. J. Can. Acad. Child Adolesc. Psychiatry 20, 265–276. 22114608 PMC3222570

[B31] Krugers H. J. Arp J. M. Xiong H. Kanatsou S. Lesuis S. L. Korosi A.. (2017). Early life adversity: lasting consequences for emotional learning. Neurobiol. Stress 6, 14–21. 10.1016/j.ynstr.2016.11.005 28229105 PMC5314442

[B32] Lajud N. Roque A. Cajero M. Gutierrez-Ospina G. Torner L. (2012). Periodic maternal separation decreases hippocampal neurogenesis without affecting basal corticosterone during the stress hyporesponsive period, but alters HPA axis and coping behavior in adulthood. Psychoneuroendocrinology 37, 410–420. 10.1016/j.psyneuen.2011.07.011 21862224

[B33] Laplante D. P. Brunet A. Schmitz N. Ciampi A. King S. (2008). Project Ice Storm: prenatal maternal stress affects cognitive and linguistic functioning in 5 1/2-year-old children. J. Am. Acad. Child Adolesc. Psychiatry 47, 1063–1072. 10.1097/CHI.0b013e31817eec80 18665002

[B34] Larsson G. Eriksson M. Zetterström R. (1979). Amphetamine addiction and pregnancy. Acta Psychiatr. Scand. 60, 334–346. 10.1111/j.1600-0447.1979.tb00283.x 517149

[B35] Lemaire V. Koehl M. Le Moal M. Abrous D. N. (2000). Prenatal stress produces learning deficits associated with an inhibition of neurogenesis in the hippocampus. Proc. Natl. Acad. Sci. U S A 97, 11032–11037. 10.1073/pnas.97.20.11032 11005874 PMC27143

[B36] Liles B. D. Newman E. Lagasse L. L. Derauf C. Shah R. Smith L. M.. (2012). Perceived child behavior problems, parenting stress and maternal depressive symptoms among prenatal methamphetamine users. Child Psychiatry Hum. Dev. 43, 943–957. 10.1007/s10578-012-0305-2 22552952 PMC3717339

[B37] Lin Y. Xu J. Huang J. Jia Y. Zhang J. Yan C.. (2017). Effects of prenatal and postnatal maternal emotional stress on toddlers’ cognitive and temperamental development. J. Affect. Disord. 207, 9–17. 10.1016/j.jad.2016.09.010 27665073

[B38] Little B. B. Snell L. M. Gilstrap L. C. III. (1988). Methamphetamine abuse during pregnancy: outcome and fetal effects. Obstet. Gynecol. 72, 541–544. 3419732

[B39] Macúchová E. Nohejlová K. Sevcíková M. Hrebícková I. Šlamberová R. (2017). Sex differences in the strategies of spatial learning in prenatally-exposed rats treated with various drugs in adulthood. Behav. Brain Res. 327, 83–93. 10.1016/j.bbr.2017.03.041 28359886

[B40] Malinová-Ševčíková M. Hrebíčková I. Macúchová E. Nová E. Pometlová M. Šlamberová R. (2014). Differences in maternal behavior and development of their pups depend on the time of methamphetamine exposure during gestation period. Physiol. Res. 4, S559–S572. 25669687 10.33549/physiolres.932925

[B41] Marais L. van Rensburg S. J. van Zyl J. M. Stein D. J. Daniels W. M. (2008). Maternal separation of rat pups increases the risk of developing depressive-like behavior after subsequent chronic stress by altering corticosterone and neurotrophin levels in the hippocampus. Neurosci. Res. 61, 106–112. 10.1016/j.neures.2008.01.011 18329744

[B42] Marwick C. (2000). NIDA seeking data on effect of fetal exposure to methamphetamine. JAMA 283, 2225–2226. 10.1001/jama.283.17.2225-jmn0503-2-1 10807367

[B43] McDonald R. J. White N. M. (1994). Parallel information processing in the water maze: evidence for independent memory systems involving dorsal striatum and hippocampus. Behav. Neural Biol. 61, 260–270. 10.1016/s0163-1047(05)80009-3 8067981

[B44] Mesquita A. R. Pego J. M. Summavielle T. Maciel P. Almeida O. F. Sousa N. (2007). Neurodevelopment milestone abnormalities in rats exposed to stress in early life. Neuroscience 147, 1022–1033. 10.1016/j.neuroscience.2007.04.007 17587501

[B45] Mirescu C. Peters J. D. Gould E. (2004). Early life experience alters response of adult neurogenesis to stress. Nat. Neurosci. 7, 841–846. 10.1038/nn1290 15273691

[B46] Morris R. G. Moser E. I. Riedel G. Martin S. J. Sandin J. Day M.. (2003). Elements of a neurobiological theory of the hippocampus: the role of activity-dependent synaptic plasticity in memory. Philos. Trans. R. Soc. Lond. B Biol. Sci. 358, 773–786. 10.1098/rstb.2002.1264 12744273 PMC1693159

[B47] Naninck E. F. Hoeijmakers L. Kakava-Georgiadou N. Meesters A. Lazic S. E. Lucassen P. J.. (2015). Chronic early life stress alters developmental and adult neurogenesis and impairs cognitive function in mice. Hippocampus 25, 309–328. 10.1002/hipo.22374 25269685

[B48] Nephew B. Murgatroyd C. (2013). The role of maternal care in shaping CNS function. Neuropeptides 47, 371–378. 10.1016/j.npep.2013.10.013 24210943 PMC3874801

[B49] Oreland S. Nylander I. Pickering C. (2010). Prolonged maternal separation decreases granule cell number in the dentate gyrus of 3-week-old male rats. Int. J. Dev. Neurosci. 28, 139–144. 10.1016/j.ijdevneu.2009.12.005 20079421

[B50] Oro A. S. Dixon S. D. (1987). Perinatal cocaine and methamphetamine exposure: maternal and neonatal correlates. J. Pediatr. 111, 571–578. 10.1016/s0022-3476(87)80125-7 3655989

[B51] Peters D. A. (1986). Prenatal stress increases the behavioral response to serotonin agonists and alters open field behavior in the rat. Pharmacol. Biochem. Behav. 25, 873–877. 10.1016/0091-3057(86)90400-4 3491370

[B52] Plotsky P. M. Thrivikraman K. V. Nemeroff C. B. Caldji C. Sharma S. Meaney M. J. (2005). Long-term consequences of neonatal rearing on central corticotropin-releasing factor systems in adult male rat offspring. Neuropsychopharmacology 30, 2192–2204. 10.1038/sj.npp.1300769 15920504

[B53] Pryce C. R. Feldon J. (2003). Long-term neurobehavioural impact of the postnatal environment in rats: manipulations, effects and mediating mechanisms. Neurosci. Biobehav. Rev. 27, 57–71. 10.1016/s0149-7634(03)00009-5 12732223

[B54] Rambousek L. Kacer P. Syslová K. Bumba J. Bubeníková-Valesova V. Šlamberová R. (2014). Sex differences in methamphetamine pharmacokinetics in adult rats and its transfer to pups through the placental membrane and breast milk. Drug Alcohol. Depend. 139, 138–144. 10.1016/j.drugalcdep.2014.03.023 24726427

[B55] Salzmann C. Otis M. Long H. Roberge C. Gallo-Payet N. Walker C. D. (2004). Inhibition of steroidogenic response to adrenocorticotropin by leptin: implications for the adrenal response to maternal separation in neonatal rats. Endocrinology 145, 1810–1822. 10.1210/en.2003-1514 14691016

[B56] Sapolsky R. M. (1985). Glucocorticoid toxicity in the hippocampus: temporal aspects of neuronal vulnerability. Brain Res. 359, 300–305. 10.1016/0006-8993(85)91440-4 4075151

[B57] Sapolsky R. M. Meaney M. J. (1986). Maturation of the adrenocortical stress response: neuroendocrine control mechanisms and the stress hyporesponsive period. Brain Res. 396, 64–76. 10.1016/0165-0173(86)90010-x 3011218

[B58] Schmidt M. V. Levine S. Alam S. Harbich D. Sterlemann V. Ganea K.. (2006). Metabolic signals modulate hypothalamic-pituitary-adrenal axis activation during maternal separation of the neonatal mouse. J. Neuroendocrinol. 18, 865–874. 10.1111/j.1365-2826.2006.01482.x 17026536

[B59] Schutová B. Hrubá L. Pometlová M. Deykun K. Šlamberová R. (2009). Cognitive functions and drug sensitivity in adult male rats prenatally exposed to methamphetamine. Physiol. Res. 58, 741–750. 19093723 10.33549/physiolres.931562

[B60] Šlamberová R. (2012). Drugs in pregnancy: the effects on mother and her progeny. Physiol. Res. 61, S123–S135. 22827869 10.33549/physiolres.932357

[B61] Šlamberová R. Charousová P. (2008). Methamphetamine—a drug of pregnant female drug addicts. Cesk Fysiol. 57, 15–23. . 18630140

[B62] Šlamberová R. Charousová P. Pometlová M. (2005). Methamphetamine administration during gestation impairs maternal behavior. Dev. Psychobiol. 46, 57–65. 10.1002/dev.20042 15633162

[B63] Šlamberová R. Pometlová M. Charousová P. (2006). Postnatal development of rat pups is altered by prenatal methamphetamine exposure. Prog. Neuropsychopharmacol. Biol. Psychiatry 30, 82–88. 10.1016/j.pnpbp.2005.06.006 16046043

[B64] Šlamberová R. Schindler C. J. Vathy I. (2002). Impact of maternal morphine and saline injections on behavioral responses to a cold water stressor in adult male and female progeny. Physiol. Behav. 75, 723–732. 10.1016/s0031-9384(02)00669-8 12020737

[B65] Smith L. M. Chang L. Yonekura M. L. Grob C. Osborn D. Ernst T. (2001). Brain proton magnetic resonance spectroscopy in children exposed to methamphetamine *in utero*. Neurology 57, 255–260. 10.1212/WNL.57.2.255 11468309

[B66] Stevenson-Hinde J. (2007). Attachment theory and John Bowlby: some reflections. Attach. Hum. Dev. 9, 337–342. 10.1080/14616730701711540 18049930

[B67] Swant J. Chirwa S. Stanwood G. Khoshbouei H. (2010). Methamphetamine reduces LTP and increases baseline synaptic transmission in the CA1 region of mouse hippocampus. PLoS One 5:e11382. 10.1371/journal.pone.0011382 20614033 PMC2894864

[B68] Thompson B. L. Levitt P. Stanwood G. D. (2009). Prenatal exposure to drugs: effects on brain development and implications for policy and education. Nat. Rev. Neurosci. 10, 303–312. 10.1038/nrn2598 19277053 PMC2777887

[B69] Vavrinková B. Binder T. Zivný J. (2001). Characteristics of a population of drug dependent pregnant women in the Czech Republic. Ceska Gynekol. 66, 285–291. 11569427

[B70] Vorhees C. V. Ahrens K. G. Acuff-Smith K. D. Schilling M. A. Fisher J. E. (1994). Methamphetamine exposure during early postnatal development in rats: I. Acoustic startle augmentation and spatial learning deficits. Psychopharmacology 114, 392–401. 10.1007/bf02249328 7855197

[B71] Walker C. D. Salzmann C. Long H. Otis M. Roberge C. Gallo-Payet N. (2004). Direct inhibitory effects of leptin on the neonatal adrenal and potential consequences for brain glucocorticoid feedback. Endocr. Res. 30, 837–844. 10.1081/erc-200044096 15666834

[B72] Williams M. T. Blankenmeyer T. L. Schaefer T. L. Brown C. A. Gudelsky G. A. Vorhees C. V. (2003). Long-term effects of neonatal methamphetamine exposure in rats on spatial learning in the Barnes maze and on cliff avoidance, corticosterone release, and neurotoxicity in adulthood. Dev. Brain Res. 147, 163–175. 10.1016/j.devbrainres.2003.11.001 14741761

[B73] Williams M. T. Schaefer T. L. Furay A. R. Ehrman L. A. Vorhees C. V. (2006). Ontogeny of the adrenal response to (+)-methamphetamine in neonatal rats: the effect of prior drug exposure. Stress 9, 153–163. 10.1080/10253890600902842 17060049 PMC2756087

[B74] Wouldes T. LaGasse L. Sheridan J. Lester B. (2004). Maternal methamphetamine use during pregnancy and child outcome: what do we know? N Z Med. J. 117:U1180. 15570349

[B75] Yamamoto Y. Yamamoto K. Fukui Y. Kurishita A. (1992). Teratogenic effects of methamphetamine in mice. Nihon Hoigaku Zasshi 46, 126–131. 1619809

[B76] Zhou L. Xu J. Zhang J. Yan C. Lin Y. Jia Y.. (2017). Prenatal maternal stress in relation to the effects of prenatal lead exposure on toddler cognitive development. Neurotoxicology 59, 71–78. 10.1016/j.neuro.2017.01.008 28159619

